# *CYP2A6* and *ERCC1* polymorphisms correlate with efficacy of S-1 plus cisplatin in metastatic gastric cancer patients

**DOI:** 10.1038/bjc.2011.24

**Published:** 2011-03-01

**Authors:** S R Park, S-Y Kong, B-H Nam, I J Choi, C G Kim, J Y Lee, S J Cho, Y W Kim, K W Ryu, J H Lee, J Rhee, Y-I Park, N K Kim

**Affiliations:** 1 Center for Gastric Cancer, Research Institute and Hospital, National Cancer Center, 111 Jungbalsan-ro, Ilsandong-gu, Goyang, Gyeonggi, 410-769, Republic of Korea; 2 Center for Clinical Services, Research Institute and Hospital, National Cancer Center, 111 Jungbalsan-ro, Ilsandong-gu, Goyang, Gyeonggi, 410-769, Republic of Korea; 3 Center for Clinical Trials, Research Institute and Hospital, National Cancer Center, 111 Jungbalsan-ro, Ilsandong-gu, Goyang, Gyeonggi, 410-769, Republic of Korea

**Keywords:** cisplatin, CYP2A6, ERCC1, gastric cancer, polymorphism, S-1

## Abstract

**Background:**

We evaluated the association between polymorphisms of cytochrome P450 2A6 (*CYP2A6*)/excision repair cross-complementation group 1 (*ERCC1*)/X-ray repair cross-complementing group 1(*XRCC1*) and treatment outcomes of metastatic gastric cancer (MGC) patients treated with S-1/cisplatin.

**Methods:**

Among MGC patients (*n*=108), who received S-1 (40 mg m^−2^ b.i.d., days 1–14) and cisplatin (60 mg m^−2^, day 1) every 3 weeks, we analysed the wild-type allele (*W*) and variants (*V*) of *CYP2A6* (*4, *7, *9, *10), and the polymorphisms of *ERCC1* (rs11615, rs3212986) and *XRCC1* (rs25487).

**Results:**

Patients having fewer *CYP2A6* variants had better response rates (*W*/*W vs W*/*V* other than *1/*4 vs V/*V* or *1/*4=66.7 *vs* 58.3 *vs* 32.3% *P*=0.008), time to progression (TTP) (7.2 *vs* 6.1 *vs* 3.5 months, *P*=0.021), and overall survival (23.2 *vs* 15.4 *vs* 12.0 months, *P*=0.004). *ERCC1 19442C*>*A* (rs3212986) was also associated with response rate (*C/C*, 46.7% *vs C/A*, 55.3% *vs A/A*, 87.5%) (*P*=0.048) and TTP (4.4 *vs* 7.6 *vs* 7.9 months) (*P*=0.012). Patients carrying both risk genotypes of *CYP2A6* (*V*/*V* or 1/*4) and *ERCC1 19442C*>*A* (*C/C*) *vs* those carrying none showed an adjusted odds ratio of 0.113 (*P*=0.004) for response, and adjusted hazard ratios of 3.748 (*P*=0.0001) for TTP and 2.961 (*P*=0.006) for death.

**Conclusion:**

Polymorphisms of *CYP2A6* and *ERCC1 19442C*>*A* correlated with the efficacy of S-1/cisplatin.

Gastric cancer is the second most common cause of cancer death worldwide and the most common cancer in Korea (Parkin *et al*, 2005; Shin *et al*, 2007). As unresectable or metastatic gastric cancer (MGC) patients have a dismal prognosis with a median survival of less than 1 year despite chemotherapy, more effective treatment is urgently needed.

Until recently, the most common combination chemotherapies for MGC were based on infusional fluorouracil (5-FU) and/or cisplatin, with cisplatin/5-FU and epirubicin/cisplatin/5-FU regarded as reference treatments. Interest in oral fluoropyrimidines has been increasing, however, because of the convenience and safety they offer, and a novel oral fluoropyrimidine, S-1 is being actively investigated. In recent phase III trial, S-1 alone, or combined with cisplatin, showed promising activity as both a palliative and as an adjuvant therapy in advanced gastric cancer (Sakuramoto *et al*, 2007; Koizumi *et al*, 2008; Boku *et al*, 2009; Ajani *et al*, 2010). Now that several phase III trials have demonstrated that both capecitabine and S-1 are not inferior to 5-FU in the setting of a platinum-containing combination, oral fluoropyrimidine plus platinum combinations are being widely used in clinical practice or as novel reference regimens in clinical trials (Cunningham *et al*, 2008; Kang *et al*, 2009; Ajani *et al*, 2010). In addition, S-1 plus cisplatin shows a better safety profile than 5-FU plus cisplatin (Ajani *et al*, 2010). On the basis of these efficacy and safety data, S-1 plus cisplatin has become one of the most commonly used regimens in MGC, yet little is known about which subset of patients is most likely to benefit from the therapy. Identification of predictive markers for efficacy and/or toxicity could lead to more tailored therapy and, ultimately, improved treatment outcomes. An oral fluoropyrimidine, S-1, consists of 5-chloro-2,4-dihydroxypyridine, potassium oxonate, and tegafur, which is converted to 5-FU in the liver mainly by cytochrome P450 2A6 (*CYP2A6*) (Shirasaka *et al*, 1996; Ikeda *et al*, 2000). The enzyme *CYP2A6* has polymorphic variants; CYP2A6*2, *4, *5, and *20 show no enzyme activity, whereas CYP2A6*6, *7, *9, *10, *11, *12, *17, *18, and *19 show reduced activity (http://www.cypalleles.ki.se). Recent pharmacokinetic studies showed the plasma concentrations and clearances of 5-FU and/or tegafur differed according to the *CYP2A6* polymorphisms in patients treated with S-1 (Fujita *et al*, 2008; Kaida *et al*, 2008; Kim *et al*, 2009). Here we hypothesise that *CYP2A6* polymorphisms affect the clinical outcomes of patients who are undergoing S-1-containing chemotherapy for MGC – poorer efficacy and/or lower toxicity in patients with defective variant alleles.

Cisplatin is cytotoxic mainly through formation of DNA adducts that cause inter- or intrastrand crosslinking. Nucleotide excision repair (NER) and base excision repair (BER) systems are involved in the repair of such damage. Key and rate-limiting enzymes include excision repair cross-complementation group 1 (ERCC1) in NER and X-ray repair cross-complementing group 1 (XRCC1) in BER, and they could lead to less cisplatin-induced DNA damage and thus to a poor drug response (Bosken *et al*, 2002; Furuta *et al*, 2002; Reed, 2005; Olaussen *et al*, 2006). Polymorphisms of those DNA repair enzymes are associated with altered functional activity and variations in clinical outcome in patients treated with cisplatin-based chemotherapy (de las Penas *et al*, 2006; Krivak *et al*, 2008; Bradbury *et al*, 2009; Kalikaki *et al*, 2009; Shim *et al*, 2010). Although there are other important factors in DNA repair pathways, we selected *ERCC1* rs11615 and rs3212986 and *XRCC1* rs25487 for genotyping, based on their frequencies of minor alleles >0.1 in Asian populations according to the Single-Nucleotide Polymorphism database (http://www.ncbi.nlm.nih.gov/projects/SNP), and previous findings of associations with treatment outcomes of gastric cancer patients treated with platinum (Liu *et al*, 2007; Goekkurt *et al*, 2009; Huang *et al*, 2009).

In this study, we investigated association between *CYP2A6*, *ERCC1*, and *XRCC1* polymorphisms and clinical outcomes of MGC patients who received S-1 plus cisplatin chemotherapy.

## PATIENTS AND METHODS

### Study population and treatment

We prospectively collected clinical data on 134 consecutive MGC patients who had received palliative S-1 plus cisplatin as first-line chemotherapy in the Center for Gastric Cancer of the National Cancer Center, Korea, between April 2006 and April 2010. Of those, we excluded 10 patients who did not have measurable lesions, 9 who were lost to follow-up during the first cycle, and 7 in whom blood samples were not available, leaving 108 patients eligible for analysis. Other eligibility criteria included Eastern Cooperative Oncology Group (ECOG) performance status ⩽2, age ⩾18 years, no concurrent uncontrolled medical illness, and adequate haematological (absolute neutrophil count ⩾1500 per *μ*l, platelet count ⩾100 000 per *μ*l), hepatic (aminotransferase ⩽2.5 × the upper limit of normal (ULN)) (⩽ 5 × ULN in the presence of liver metastases), total bilirubin ⩽1.5 × ULN), and renal (creatinine ⩽1.5 × ULN) function.

Treatment consisted of 40 mg m^−2^ oral S-1 twice daily (within the hour following morning and evening meals) on days 1 to 14, and a 15-min intravenous infusion of cisplatin 60 mg m^−2^ on day 1 of a 3-week cycle. Prophylactic administration of granulocyte-colony stimulating factor was not allowed. To prevent nausea and vomiting, 5-hydroxytryptamine-3 receptor antagonists and dexamethasone and/or aprepitant were administered before chemotherapy. Treatment was continued in the absence of disease progression or unacceptable toxicity.

All patients provided written informed consent, and the study was approved by the Institutional Review Board at the Research Institute and Hospital, National Cancer Center. All information was obtained with appropriate Institutional Review Board waivers.

### Assessment of efficacy and toxicity

Computed tomography scans were performed every 2–3 cycles or if clinically indicated to evaluate tumour response to treatment, which was assessed according to Response Evaluation Criteria in Solid Tumors (Therasse *et al*, 2000). Objective responses were confirmed by a second evaluation 4 to 6 weeks later. The response rate and time to progression (TTP) were assessed by investigators. A complete blood cell count with differential, serum chemistry profile, and electrolyte level analysis were performed every 3 weeks. Toxicity was graded according to National Cancer Institute Common Toxicity Criteria (version 3.0).

### *CYP2A6*, *ERCC1*, and *XRCC1* genotyping

Using genomic DNA extracted from 3 ml peripheral blood with a Qiagen DNA extraction kit (Qiagen, Hilden, Germany), we identified the common variant alleles that affect CYP2A6 activity or expression in Asian populations (CYP2A6*4, *7, *9, and *10), as well as the wild-type allele (CYP2A6*1), as previously described (Kong *et al*, 2009). Briefly, we used polymerase chain reaction (PCR)–restriction fragment length polymorphism, sequencing, and primer extension methods to determine three polymorphic sites (–*48T*>*G*, *6558T*>*C*, and *6600G*>*T*) and deletion of the *CYP2A6* gene. We used a GeneAmp PCR system 9700 thermal cycler (Applied Biosystems, Foster City, CA, USA) and performed electrophoresis with an ABI Prism 3100 analyser (Applied Biosystems).

We genotyped *ERCC1* polymorphisms, including rs11615 {NG_015839.1}: g.*8525C*>*T* p.*Asn118Asn*) and rs3212986 {NG_015839.1}:g.*19442C*>*A*, at 3^′^ untranslated region), using the TaqMan SNP assay (Applied Biosystems) following the manufacturer′s directions. We performed sequence analysis for rs25487 of the *XRCC1* polymorphism ({NM_006297.2}:c.*1196G*>*A*, p.*Arg399Gln*), using the PCR product, which was reacted with a mixture of HF taq premix (Bioneer, Daejeon, Korea), primers (F: 5^′^-CCTCTCTCGTTCCCCTTTG-3^′^ and R: 5^′^-AGGTCCTCCTTCCCTCATCT-3^′^), and sample DNA.

### Statistical analysis

We assessed associations between variables using the Pearson *χ*^2^-test or the Fisher's exact test for categorical variables. We performed multivariate logistic regression analyses to ascertain whether the genetic polymorphisms are independently associated with treatment responses after adjusting for other relevant variables. We defined TTP as the time from the initiation of treatment to the date of documented disease progression, and defined overall survival (OS) as the time from the initiation of treatment to the date of death from any cause or the last follow-up visit. We used the Kaplan–Meier method and the log-rank test to estimate and compare survival distribution, and used Cox-regression models for survival multivariate analysis. We used trend tests to assess statistical significance of changes in the relationship between treatment efficacy and genetic polymorphism. We categorised genetic polymorphisms on an ordinal scale, according to genotypes (0 for homozygous non-risk allele, 1 for heterozygous risk allele, and 2 for homozygous risk allele), with risk alleles defined as those associated with poorer treatment efficacy. We considered the results as statistically significant when two-sided *P*-values were <0.05.

## RESULTS

### Patient characteristics

Table 1 shows the clinical characteristics of the 108 eligible patients. Their median age was 57 years (range, 26–72), and the median follow-up period was 19.9 months (range, 1.4–59.1 months). Most (88.9%) of the patients had an ECOG performance status of 0–1, and all had metastatic disease, with 24 (22.2%) of them having recurrent metastatic disease after previous curative gastrectomy. Among the characteristics listed in Table 1, ECOG performance status (0–1 *vs* 2) was significantly associated with tumour response rate (Pearson *χ*^2^-test, *P*=0.008), TTP (log-rank *P*<0.001), and OS (log-rank *P*<0.001); sex was significantly associated with tumour response rate (Pearson *χ*^2^-test, *P*=0.004) and TTP (log-rank *P*=0.041); and number of organs with metastases (<3 *vs* ⩾3) was significantly associated with TTP (*P*=0.029).

### Genotype frequencies

Table 2 shows the frequencies of the various genotypes. The allelic frequencies were 0.57 for CYP2A6*1, 0.13 for CYP2A6*4, 0.07 for CYP2A6*7, 0.20 for CYP2A6*9, and 0.03 for CYP2A6*10, and were comparable to those previously reported in Asian populations (Kwon *et al*, 2001; Schoedel *et al*, 2004; Mwenifumbo *et al*, 2005; Nakajima *et al*, 2006). To analyse the effect of the *CYP2A6* polymorphisms on treatment outcomes, we classified CYP2A6*4, CYP2A6*7, CYP2A6*9, and CYP2A6*10 as variant alleles. We assigned patients who carried *1/*7, *1/*9, or *1/*10 to a wild-type/variant (*W*/*V*) group and those who carried two variant alleles to a variant/variant (*V*/*V*) group. However, because CYP2A6*4 leads to deletion of the entire gene and thus loss of enzyme activity (patients homo- or heterozygous for the *4 allele might have lower enzyme activity or expression than those with other allele variants), we sorted the genotypes into three groups – *W/W*, *W/V* other than *1/*4, and *V/V* or *1/*4.

The frequencies were 0.76 and 0.24 for the *C* and *T* alleles, respectively, of rs11615 (*Asn118Asn*) of *ERCC1*, 0.75 and 0.25 for the *C* and *A* alleles at *19442C*>*A* of *ERCC1* (rs3212986), and 0.63 and 0.37 for the *G* and *A* alleles of rs25487 (*Arg399Gln*) of *XRCC1*. We found no significant association between any polymorphisms and age, sex, ECOG performance status, disease status, histology, tumour location, or number of organs with metastases (data not shown).

### Association between genotype and tumour response

Two of the eligible patients were lost to follow-up after the first chemotherapy cycle, leaving 106 patients, who could be evaluated for tumour response. Of those, 56 (52.8%) achieved partial response, 28 (26.4%) had stable disease, and 22 (20.8%) showed disease progression. The objective tumour response rate was 52.8% (95% confidence interval (CI), 43.3–62.3). In univariate analysis, the *CYP2A6* genotype was significantly associated with tumour response rates; 66.7% for *W/W* patients *vs* 58.3% for *W/V* other than *1/*4 vs 32.3% for *V/V* or *1/*4 (Pearson *χ*^2^-test, *P*=0.019; trend test, *P*=0.008) (Table 3). In multivariate logistic regression analysis, the *CYP2A6* genotype was significantly associated with the tumour response after adjustment for ECOG performance status and sex. The adjusted odds ratios (ORs) of patients with *W/V* other than *1/*4, and those with *V/V* or *1/*4, relative to patients with *W/W*, were 0.771 (95% CI, 0.262–2.268; *P*=0.636) and 0.220 (95% CI, 0.067–0.719; *P*=0.012), respectively (Table 4). *ERCC1 19442C*>*A* was also associated with tumour response rate in univariate analysis (*C/C*, 46.7% *vs C/A*, 55.3% *vs A/A*, 87.5%) (Fisher’s exact test, *P*=0.093; trend test, *P*=0.048) (Table 3). When we investigated the combined effect of *CYP2A6* and *ERCC1 19442C*>*A* genotypes on response rate by classifying patients into three groups according to number of risk genotypes (0, 1, or 2) for *V/V* or 1/*4, and *C/C*, we found that the response rate went down, as the number of risk genotypes went up, and the association was significant (63.6% (95% CI, 47.2–80.0) for group with 0 risk genotype; 58.2% (95% CI, 45.2–71.2) for group with one; and 16.7% (95% CI, 0–33.9) for group with both) (Pearson *χ*^2^-test, *P*=0.003; trend test, *P*=0.004) (Table 3). Adjusted ORs for tumour response for patients carrying one and two risk genotypes, compared with those carrying none, were 0.814 (95% CI, 0.308–2.151; *P*=0.678) and 0.113 (95% CI, 0.025–0.509; *P*=0.004), respectively (Table 4). Other genotypes showed no association with response rate (Table 3).

### Association between genotypes and TTP and OS

**Figure 1:**
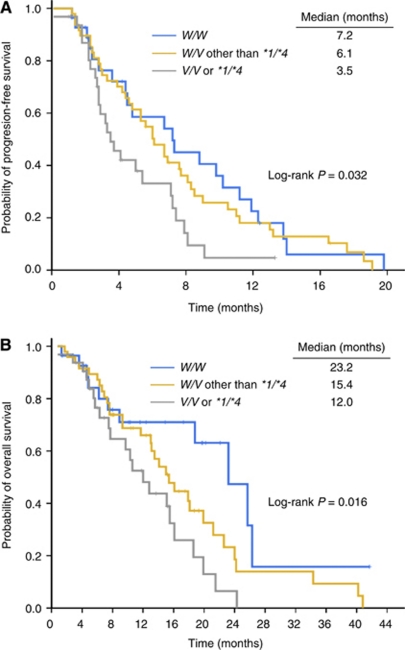
Kaplan–Meier curves of (**A**) time to progression and (**B**) overall survival according to *CYP2A6* genotype. *W*=wild-type allele; *V*=variant allele (CYP2A6*4, *7, *9, or *10).

Time to progression varied significantly with *CYP2A6* genotype (median TTP, 7.2 months for *W/W* patients *vs* 6.1 months for *W/V* other than *1/*4 vs 3.5 months for *V/V* or *1/*4) (log-rank *P*=0.032; trend test *P*=0.021) (Table 3, Figure 1A), and the *CYP2A6* genotype was a significant independent risk factor for TTP after adjustment for ECOG performance status, sex, and number of organs with metastases (Table 4). The adjusted hazard ratios (HRs) of patients with *W/V*, other than *1/*4, and those with *V/V* or *1/*4, relative to patients with *W/W* were 1.165 (95% CI, 0.686–1.981; *P*=0.572) and 2.288 (95% CI, 1.245–4.207; *P*=0.008), respectively (Table 4). Overall survival also varied significantly with *CYP2A6* genotype (median OS, 23.2 months for *W/W* patients *vs* 15.4 months for *W/V* other than *1/*4 vs 12.0 months for *V/V* or *1/*4) (log-rank *P*=0.016; trend test *P*=0.004) (Table 3, Figure 1B), and the *CYP2A6* genotype was a significant independent predictor for OS after adjustment for ECOG performance status. The adjusted HRs of patients with *W/V* other than *1/*4 and those with *V/V* or *1/*4, relative to patients with *W/W*, were 1.806 (95% CI, 0.906–3.598; *P*=0.093) and 3.118 (95% CI, 1.483–6.558; *P*=0.003), respectively (Table 4).

**Figure 2:**
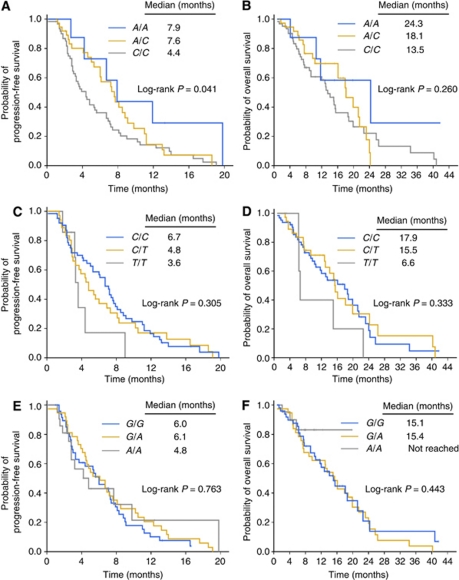
Kaplan–Meier curves of (**A**) time to progression and (**B**) overall survival according to *ERCC1 19442C*>*A* (rs3212986) genotype, (**C**) time to progression, (**D**) overall survival according to *ERCC1 8525C*>*T* (rs11615) genotype, (**E**) time to progression, and (**F**) overall survival according to *XRCC1 1196G*>*A* (rs25487) genotype.

Among the *ERCC1* and *XRCC1* polymorphisms, *ERCC1 19442C*>*A* was significantly associated with TTP, with risk in the order of *C/C*>*C/A*>*A/A* (median TTP, 4.4 months for *C/C* patients *vs* 7.6 months for *C/A vs* 7.9 months for *A/A*) (log-rank *P*=0.041; trend test *P*=0.012) (Table 3, Figure 2A). In multivariate analysis, the *ERCC1 19442C*>*A* genotype was an independent predictor for TTP with borderline significance after adjustment for ECOG performance status, sex, and number of organs with metastases (adjusted HR for patients carrying *C/C* compared with patients carrying *A/A* genotype as a reference, was 2.365 (95% CI, 0.909–6.155; *P*=0.078) (Table 4). The adjusted HR of patients with *A/A* or *A/C* relative to patients with *C/C* was 1.546 (95% CI, 0.990–2.413; *P*=0.055).

**Figure 3:**
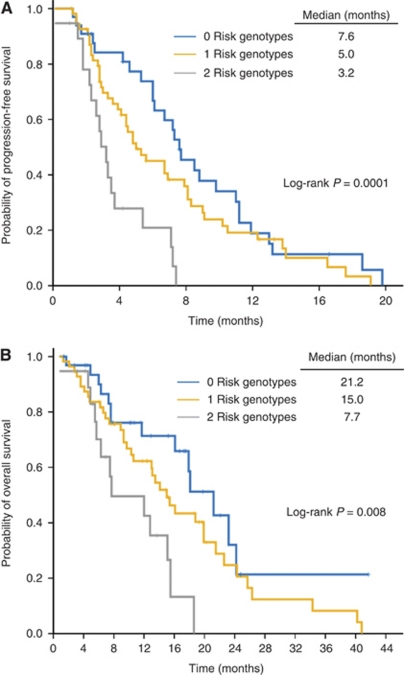
Kaplan–Meier curves of (**A**) time to progression and (**B**) overall survival according to the number of risk genotypes of *CYP2A6* (*V/V* or 1/*4) and *ERCC1 19442C*>*A* (*C/C*).

The combination of *CYP2A6* and *ERCC1 19442C*>*A* genotypes showed the number of risk genotypes had cumulative effects on TTP (median TTP, 7.6 months (95% CI, 5.979–9.221) for group with 0 risk genotype; 5.0 months (95% CI, 2.971–7.029) for group with one; and 3.2 months (95% CI, 2.376–4.024) for group with both) (log-rank *P*=0.0001; trend test, *P*=0.0005) (Table 3, Figure 3A). Adjusted HRs for disease progression for patients carrying one and two risk genotypes were 1.396 (95% CI, 0.851–2.290; *P*=0.187) and 3.748 (95% CI, 1.900–7.393; *P*=0.0001), respectively (Table 4). Similarly, the number of risk genotypes had cumulative effects on OS (median OS, 21.2 months (95% CI, 16.1–26.3) for group with 0 risk genotype; 15.0 months (95% CI, 11.8–18.2) for group with one; and 7.7 months (95% CI, 0–15.7) for group with both) (log-rank *P*=0.008; trend test, *P*=0.007) (Table 3, Figure 3B). Adjusted HRs for death for patients carrying one and two risk genotypes were 1.374 (95% CI, 0.750–2.517; *P*=0.303) and 2.961 (95% CI, 1.371–6.393; *P*=0.006), respectively (Table 4).

### Association between genotypes and toxicity

The *CYP2A6* polymorphisms showed no significant association with grade 3 or 4 haematological toxicity during the first cycle (leukopenia, *P*=1.000; neutropenia, *P*=0.784; anaemia, *P*=0.353; thrombocytopenia, *P*=0.169). Nor was there an association between *ERCC1 19442C*>*A* genotypes and grade 3 or 4 haematological toxicity during the first cycle (leukopenia, *P*=0.273; neutropenia, *P*=0.290; anaemia, *P*=1.000; thrombocytopenia, *P*=1.000). The same was true for *ERCC1 8525C*>*T (*leukopenia, *P*=1.000; neutropenia, *P*=0.909; anaemia, *P*=0.310; thrombocytopenia, *P*=0.625), and for *XRCC1* variation (leukopenia, *P*=0.672; neutropenia, *P*=0.600; anaemia, *P*=0.158; thrombocytopenia, *P*=0.590). We also found no significant association between any genotype and non-haematological toxicity during the first cycle (data not shown).

## DISCUSSION

In this retrospective study of MGC patients given S-1 plus cisplatin as first-line chemotherapy, we demonstrated that *CYP2A6* and *ERCC1 19442C*>*A* polymorphisms correlated with treatment efficacy, and that *CYP2A6* polymorphism was an especially strong independent predictor for all efficacy endpoints – response rate, TTP, and OS. Response, TTP, and OS were significantly poorer in patients with two variant alleles or *1/*4 whose enzyme product has reduced or no activity and that could result in a reduced conversion rate of tegafur to 5-FU. Our finding that patients with a *V/V* or *1/*4 genotype compared with those with a *W/W* genotype had a probability of response of only 0.22 and a 2.29-fold risk of progression, and a 3.12-fold risk of death are consistent with finding in MGC patients treated with S-1 plus docetaxel (Kong *et al*, 2009). In addition, our present results are supported by the findings that CYP2A6*4 reduces plasma 5-FU concentration and increases the area of under the concentration-time curve (AUC) and *C*_max_ for tegafur in non-small cell lung cancer patients treated with S-1 alone or in combination with cisplatin (Kaida *et al*, 2008). Moreover, in advanced biliary cancer patients, treated with S-1 plus oxaliplatin, the AUC and *C*_max_ for 5-FU and the metabolic ratio (exposure ratio of 5-FU to tegafur) are significantly higher in patients homozygous for wild-type *CYP2A6* than in those with one or two variant alleles (*4, *7, or *9) (Kim *et al*, 2009), again suggesting that *CYP2A6* genotype is a strong predictor of the efficacy of S-1-based chemotherapy.

Another predictor that has been suggested is tumour tissue expression level of mRNA or protein of 5-FU metabolic pathway genes, such as *thymidylate synthase*, *orotate phosphoribosyltransferase*, and *thymidine phosphorylase* (Ichikawa *et al*, 2006; Choi *et al*, 2010; Koizumi *et al* 2010). Results from those studies, however, have not been consistent. In addition, genotyping peripheral blood cells, which we did in the present study, is more optimal than evaluating tumour expression levels of mRNA or protein, in which there may not be clinical accessibility or the assay may not be applicable because of arbitrary cutoff levels, lack of standard criteria, or observer variation.

In the current study, we categorised *CYP2A6* polymorphism into three groups (*W/W vs W/V* other than *1/*4 vs V/V or *1/*4) according to the CYP2A6 enzyme activity. As CYP2A6*4 allele and the other alleles do not have the same enzyme activity – CYP2A6*4 allele causes a *CYP2A6* gene deletion, which lacks activity and CYP2A6*7, *9, and *10 cause the decreased enzymatic activity – we combined these *7, *9, and *10 defective alleles into a variant allele, but differentiated *4 from other variant alleles. Previous studies have shown that the serum nicotine/cotinine ratio (nicotine is metabolised primarily by CYP2A6 by C-oxidation to cotinine) was higher in the subjects with CYP2A6*4/*4, *7/*7, or *9/*9 rather than in those with CYP2A6*1/*1, and that the inhibitory effects of CYP2A6*7 on nicotine metabolism were comparable to those by CYP2A6*9 (Yoshida *et al*, 2002, 2003). In *in vivo* measurement of nicotine oxidation, CYP2A6*4/*7 and CYP2A6*4/*10 demonstrated similar enzyme activity to CYP2A6*4/*4 (Xu *et al*, 2002). The cotinine/nicotine ratios (±s.d.) in the subjects with CYP2A6*9/*9 (4.3±2.4) were significantly lower than those in the subjects with CYP2A6*1/*9 (7.7±5.6) and CYP2A6*1/*1 (10.4±9.2) (Yoshida *et al*, 2003). The subjects with CYP2A6*1/*4 had the lower cotinine/nicotine ratios (4.79±3.17) than either those with CYP2A6*1/*1 (7.42±6.56), or with CYP2A6*1/*7 (6.27±4.76) (Yoshida *et al*, 2002). In addition, the mean *in vitro* coumarin 7-hydroxylase activities, which are catalysed by CYP2A6, in subjects carrying CYP2A6*1/*4, CYP2A6*1/*9, and CYP2A6*4/*9 were 41, 71, and 12%, respectively, compared with those of the subjects with wild-type alleles (Kiyotani *et al*, 2003). Furthermore, when patients were classified into three groups (group 1=wild-type *vs* group 2=CYP2A6*1/*9 or ^*^1/*12 vs group 3=CYP2A6*1/*2, *1/*4, *9/*12, *9/*4, or *9/*9), the fractional clearance of nicotine to cotinine was about 80% in group 2, and about 50% in group 3 compared with group 1 (Benowitz *et al*, 2006). The mean total plasma clearance of nicotine was 18.8±6.0, 15.5±4.9, and 11.7±5.1 ml min^−1^ kg^−1^ in groups 1, 2, and 3, respectively. These data suggest that CYP2A6*1/*4 results in lower enzyme activity than other *W/V* genotypes.

Although *ERCC1 19442C*>*A* was significantly associated with response rate, the exact functional consequences of this polymorphism have yet to be elucidated. The 3^′^ untranslated region might affect mRNA stability and result in lower expression levels of the enzyme (Chen *et al*, 2000); the lower DNA repair capacity would increase the damage done by platinum agents and hence, increase their efficacy. In clinical studies, however, the *ERCC1 19442C*>*A* polymorphism has provided variable results. Our finding that the *C/C* genotype, compared with the *C/A* or *A/A* genotype, was associated with poorer treatment efficacy is consistent with studies of cisplatin-treated oesophageal or non-small cell lung cancer patients (Bradbury *et al*, 2009; Kalikaki *et al*, 2009). In contrast, other studies reported that the *A* allele was associated with shorter progression-free survival and/or OS in stage III non-small cell lung cancer or epithelial ovarian cancer patients treated with platinum-based chemotherapy (Zhou *et al*, 2004; Krivak *et al*, 2008). These mixed results might be attributable to variation in patient or tumour characteristics and treatments delivered across studies.

Our finding that the number of risk genotypes of *CYP2A6* and *ERCC1 19442C*>*A* (*V/V* or *1/*4 and *C/C*) was significantly associated with treatment efficacy, and especially, the group with both showed very poor clinical outcomes (response rate 16.7%, median TTP 3.2 months, and median OS 7.7 months) suggests that *CYP2A6* polymorphisms predict primarily efficacy of S-1-based chemotherapy and *ERCC1 19442C*>*A* polymorphisms have an additive predictive role in the S-1 plus cisplatin setting. Further investigation is warranted to determine whether patients with *CYP2A6* and *ERCC1 19442C*>*A* risk genotypes would have better outcomes if they were treated with fluoropyrimidines that do not require *CYP2A6* activation, such as 5-FU itself or capecitabine, and/or non-platinum agents.

The present study had the following limitations: (1) Although we collected clinical data prospectively, this was a retrospective analysis, so our findings should be validated in prospective studies; (2) As pharmacokinetic data were not available, we could not evaluate the association between *CYP2A6* genotype and the pharmacokinetic parameters of S-1 components and their active metabolites; (3) As the current study is not a prospective, randomised trial, it cannot be excluded that *CYP2A6* gene polymorphism could be a prognostic factor of gastric cancer independent of the effect of S-1; (4) We had no functional data for the *ERCC1 19442C*>*A* polymorphisms; (5) We did not analyse polymorphisms in other genes, such as *thymidylate synthase* that might influence 5-FU efficacy.

In conclusion, our study demonstrated that the *CYP2A6* and *ERCC1 19442C*>*A* genotypes correlated with the efficacy of S-1 plus cisplatin in MGC patients. These results are consistent with the findings of our previous study, which reported for the first time that the *CYP2A6* genotype was associated with the treatment efficacy of S-1 plus docetaxel in MGC patients (Kong *et al*, 2009). Large-scale randomised prospective studies are warranted to validate our findings, which might provide useful information for selecting appropriate candidates for S-1-based chemotherapy, ultimately leading to a more tailored approach to chemotherapy.

## Figures and Tables

**Table 1:** Patient characteristics (*n*=108)

**Characteristic**	**No. of patients**	**%**
*Gender*		
Male	74	68.5
Female	34	31.5
		
Median age, years (range)	57 (26–72)	
		
*ECOG performance status*		
0	3	2.8
1	93	86.1
2	12	11.1
		
*Tumour histology*		
W/D or M/D adenocarcinoma	30	27.8
P/D, signet ring cell, or mucinous carcinoma	78	72.2
		
*Tumour location in stomach*		
Upper one third	17	15.7
Lower two thirds	70	64.8
Whole stomach	21	19.4
		
*Organ with metastases*		
Peritoneum	67	62.0
Abdominal distant lymph node	37	34.3
Liver	30	27.8
Bone or lung	12	11.1
Others	40	37.0
		
*No. of organs with metastases*		
1	49	45.4
2	38	35.2
⩾3	21	19.4
		
*Disease status*		
Initial metastatic	84	77.8
Recurrent	24	22.2
		
*Prior treatment*		
Total gastrectomy	13	12.0
Subtotal gastrectomy	27	25.0
Palliative gastrojejunostomy	2	1.9
Adjuvant chemotherapy	12	11.1

Abbreviations: ECOG=Eastern Cooperative Oncology Group; M/D=moderately differentiated; P/D=poorly differentiated; W/D=well differentiated.

**Table 2:** Genotype frequency

**Genotype**	**No. of patients**	**%**
CYP2A6		
W/W		
*1/*1	28	25.9
W/V other than *1/*4		
*1/*7	12	11.1
*1/*9	32	29.6
*1/*10	4	3.7
V/V or *1/*4		
*1/*4	19	17.6
*4/*7	3	2.8
*4/*9	5	4.6
*4/*10	2	1.9
*9/*9	3	2.8
		
ERCC1 (rs11615) 8525C>T		
* C/C*	64	59.3
* C/T*	37	34.3
* T/T*	7	6.5
		
ERCC1(rs3212986) 19442C>A		
* C/C*	62	57.4
* C/A*	38	35.2
* A/A*	8	7.4
XRCC1 (rs25487) *1196G*>*A*		
* G/G*	49	45.4
* G/A*	38	35.2
* A/A*	21	19.4

Abbreviations: *V*=variant allele that abolishes or reduces CYP2A6 activity (*4, *7, *9, and *10); *W*=wild-type allele of CYP2A6 (*1).

**Table 3:** Univariate analysis of association between genotypes and RR, TTP, and OS

**Genotype**	**No. (%)**	**RR (%)**	***P*-value^a^**	**OR (95% CI)**	***P*-value**	**TTP (months) (95% CI)**	***P*-value^a^**	**HR (95% CI)**	***P*-value**	**OS (months) (95% CI)**	***P*-value^a^**	**HR (95% CI)**	***P*-value**
CYP2A6			0.008				0.021				0.004		
*W/W*	28 (26)	66.7		1 (reference)		7.2 (2.7–11.7)		1 (reference)		23.2 (16.3–30.1)		1 (reference)	
*W/V* other than *1/*4	48 (44)	58.3		0.699 (0.262–1.873)	0.478	6.1 (4.6–7.6)		1.168 (0.689–1.978)	0.565	15.4 (12.0–18.8)		1.759 (0.884–3.500)	0.108
*V/V* or ^*^1/*4	32 (30)	32.3		0.238 (0.079–0.714)	0.010	3.5 (2.3–4.7)		2.059 (1.130–3.751)	0.018	12.0 (8.2–15.8)		2.836 (1.350–5.957)	0.006
													
ERCC1 8525C>T			0.657				0.200				0.530		
*C/C*	64 (59)	54.0		1 (reference)		6.7 (5.5–7.9)		1 (reference)		17.9 (11.2–24.6)		1 (reference)	
*T/C*	37 (34)	52.8		0.953 (0.420–2.164)	0.909	4.8 (3.1–6.5)		1.144 (0.732–1.788)	0.556	15.5 (13.3–17.7)		0.940 (0.556–1.587)	0.816
*T/T*	7 (6)	42.9		0.640 (0.132–3.096)	0.579	3.6 (3.2–4.0)		1.916 (0.810–4.532)	0.139	6.6 (6.0–7.2)		1.918 (0.748–4.919)	0.175
													
ERCC1 19442C>A			0.048				0.012				0.109		
*A/A*	8 (7)	87.5		1 (reference)		7.9 (4.9–10.9)		1 (reference)		24.3 (5.2–43.4)		1 (reference)	
*C/A*	38 (35)	55.3		0.176 (0.020–1.577)	0.121	7.6 (5.6–9.6)		1.804 (0.691–4.710)	0.228	18.1 (13.9–22.3)		1.829 (0.608–5.504)	0.283
*C/C*	62 (57)	46.7		0.125 (0.014–1.080)	0.059	4.4 (3.2–5.6)		2.648 (1.050–6.675)	0.039	13.5 (11.0–16.0)		2.242 (0.793–6.338)	0.128
													
XRCC1 1196G>A			0.947				0.489				0.683		
*G/G*	49 (45)	51.1		1 (reference)		6.0 (4.3–7.7)		1 (reference)		15.1 (10.8–19.4)		1 (reference)	
*G/A*	38 (35)	57.9		1.317 (0.557–3.115)	0.530	6.1 (3.8–8.4)		0.863 (0.546–1.364)	0.528	15.4 (11.9–18.9)		1.137 (0.681–1.897)	0.623
*A/A*	21 (19)	47.6		0.871 (0.311–2.439)	0.793	4.8 (1.9–7.7)		0.829 (0.431–1.597)	0.576	NR		0.532 (0.159–1.776)	0.305
*No. of risk genotypes of* CYP2A6 and ERCC1 19442C>A^b^			0.004				0.0005				0.007		
0	33 (31)	63.6		1 (reference)		7.6 (6.0–9.2)		1 (reference)		21.2 (16.1–26.3)		1 (reference)	
1	56 (52)	58.2		0.795 (0.327–1.934)	0.613	5.0 (3.0–7.0)		1.391 (0.856–2.261)	0.182	15.0 (11.8–18.2)		1.432 (0.782–2.619)	0.245
2	19 (18)	16.7		0.114 (0.027–0.477)	0.003	3.2 (2.4–4.0)		3.693 (1.924–7.088)	0.00008	7.7 (0–15.7)		3.067 (1.424–6.604)	0.004
													

Abbreviations: HR=Hazards ratio; OR=odds ratio; OS=overall survival; RR=response rate; TTP=time to progression; *V*=variant allele that abolishes or reduces CYP2A6 activity (*4, *7, *9, and *10); *W*=wild-type allele of CYP2A6 (*1).

a Trend test.

b At-risk genotypes: *V/V* or 1/*4 for *CYP2A6* and *C/C* for *ERCC1 19442C*>*A*.

**Table 4:** Multivariate analysis of association between genotype and RR, TTP, and OS

	**RR**		**TTP**		**OS**	
**Genotype**	**Adjusted OR^a^ (95% CI)**	**P-value**	**Adjusted HR^b^ (95% CI)**	**P-value**	**Adjusted HR^c^ (95% CI)**	**P*-*value**
CYP2A6						
*W/W*	1 (reference)		1 (reference)		1 (reference)	
*W/V* other than *1/*4	0.771 (0.262–2.268)	0.636	1.165 (0.686–1.981)	0.572	1.806 (0.906–3.598)	0.093
*V/V* or *1/*4	0.220 (0.067–0.719)	0.012	2.288 (1.245–4.207)	0.008	3.118 (1.483–6.558)	0.003
						
ERCC1 19442C>A						
*A/A*	1 (reference)		1 (reference)		1 (reference)	
*A/C*	0.258 (0.027–2.451)	0.238	1.638 (0.616–4.357)	0.322	1.750 (0.578–5.304)	0.322
*C/C*	0.192 (0.021–1.748)	0.143	2.365 (0.909–6.155)	0.078	1.988 (0.697–5.670)	0.199
						
*No. of risk genotypes of* CYP2A6 *and* ERCC1 19442C>*A*^d^						
0	1 (reference)		1 (reference)		1 (reference)	
1	0.814 (0.308–2.151)	0.678	1.396 (0.851–2.290)	0.187	1.374 (0.750–2.517)	0.303
2	0.113 (0.025–0.509)	0.004	3.748 (1.900–7.393)	0.0001	2.961 (1.371–6.393)	0.006

Abbreviations: HR=Hazards ratio; OR=odds ratio; OS=overall survival; RR=response rate; TTP=time to progression; *V*=variant allele that abolishes or reduces CYP2A6 activity (*4, *7, *9, and *10); *W*=wild-type allele of CYP2A6 (*1).

a Adjusted for ECOG performance status (0–1 *vs* 2) and sex.

b Adjusted for ECOG performance status (0–1 *vs* 2), sex, and number of organ with metastases (<3 *vs* ⩾3).

c Adjusted for ECOG performance status (0–1 *vs* 2).

d At-risk genotypes: *V/V* or 1/*4 for *CYP2A6* and *C/C* for *ERCC1 19442C*>*A*.
